# Glutamate-Cysteine Ligase Catalytic Subunit Attenuated Hepatitis C Virus-Related Liver Fibrosis and Suppressed Endoplasmic Reticulum Stress

**DOI:** 10.3389/fmolb.2020.00199

**Published:** 2020-08-18

**Authors:** Na Fu, Dongdong Li, Wencong Li, Wen Zhao, Siyu Zhang, Lingdi Liu, Suxian Zhao, Jinghua Du, Lingbo Kong, Rongqi Wang, Yuguo Zhang, Yuemin Nan

**Affiliations:** ^1^Department of Traditional and Western Medical Hepatology, Third Hospital of Hebei Medical University, Shijiazhuang, China; ^2^Hebei Provincial Key Laboratory of Liver Fibrosis in Chronic Liver Diseases, Shijiazhuang, China

**Keywords:** glutamate-cysteine ligase catalytic subunit, hepatitis C virus, liver fibrosis, hepatic stellate cells, endoplasmic reticulum stress, oxidative stress

## Abstract

The study aimed to clarify the role and molecular mechanism of glutamate-cysteine ligase catalytic subunit (GCLC) in modulating Hepatitis C virus (HCV)-related liver fibrosis. Twenty patients with HCV-related liver fibrosis and 15 healthy controls were enrolled. Differentially expressed plasma mRNAs were detected by digital gene expression profile analysis and validated by qRT-PCR. Hepatic histopathology was observed by H&E and Masson stained liver sections. The mRNA and protein expression of GCLC, endoplasmic reticulum (ER) stress markers, and inflammatory and fibrogenic factors were detected in liver tissues from patients with HCV-related hepatic fibrosis and HCV core protein-expressing LX-2. The GCLC-overexpressing LX-2 were established by transfecting puc19-GCLC plasmid. Then, glutathione and reactive oxygen species (ROS) levels were measured respectively by spectrophotometric diagnostic kit and dihydrodichlorofluorescein diacetate kit. GCLC were dramatically down-regulated in HCV-related fibrotic livers and activated HSCs, which companied with up-regulation of ER stress-related genes, including inositol-requiring 1 (IRE1) and glucose-regulated protein 78 (GRP78). Also, the proinflammatory and profibrogenic gene, including nuclear factor kappa B (NF-κB), tumor necrosis factor α (TNFα), and transforming growth factor 1(TGFβ1), was highly upregulated. Overexpression of GCLC in hepatic stellate cells could suppress α-SMA and collagen I expression, produce hepatic GSH and reduce ROS, and down-regulate IRE1, GRP78, NF-κB, TNF-α, and TGFβ1 expression. GCLC was a negative regulatory factor in the development of HCV-related liver fibrosis and might be a potential therapeutic target for liver fibrosis.

## Introduction

Hepatitis C virus (HCV) infection is an important etiology of chronic liver disease. Approximately 185 million people were infected HCV worldwide, and an estimated 71 million people had chronic HCV infection, eventually leading to liver fibrosis, cirrhosis, and even hepatocellular carcinoma (HCC) (Pradat et al., 2018), which was the main cause of death of HCV patients (Lanini et al., 2016). With recent advancements of direct-acting antiviral (DAA) treatment, which could efficiently control viremia, however, there are still very few treatment strategies available for preventing the pathogenesis in advanced liver fibrosis or cirrhosis in patients (Khatun and Ray, 2019). Multiple possible molecular mechanisms were involved in the progression of liver fibrosis in chronically HCV-infected patients (Park et al., 2018; Khatun and Ray, 2019). Therefore, the further revealing novel genes for regulating liver fibrosis might provide evidence for gene diagnosis and molecular-targeted therapy.

In this study, we examined the differentially expressed mRNA in plasma samples from the healthy control and patients with HCV-related liver fibrosis. Our data suggested that aberrant mRNAs might contribute to the progression of HCV-related hepatic fibrosis, among that glutamate-cysteine ligase catalytic subunit (GCLC) was significantly differentially expressed. GCLC was a subunit of glutamate-cysteine ligase (GCL), which was the rate-limiting enzyme for GSH synthesis (Lu, 2013). And GSH had been reported to protect against oxidative stress (Espinosa-Díez et al., 2017) and maintain the quiescent status of hepatic stellate cells (HSCs) (Ramani et al., 2012), whose activation played a central role in liver fibrosis (Zhang et al., 2016; Huang et al., 2017). However, the role and potential mechanism of GCLC in HCV-related liver fibrosis remained largely unknown. Therefore, the aim of this study was to clarify the role and potential mechanism of GCLC in the activation of HSCs and hepatic fibrosis induced by HCV infection.

## Materials and Methods

### Patients

Twenty HCV patients with moderate to severe liver fibrosis and 15 age- and gender-matched healthy subjects, all from the Third Hospital of Hebei Medical University (Shijiazhuang, China), were included in this study. The demographic characteristics of the study subjects were summarized in Table 1. Chronic HCV infection and stage of liver fibrosis was confirmed using serology and hepatic pathology. Written informed consents were obtained prior to sample collection. The study was approved by the Human Ethics Committee, the Third Hospital of Hebei Medical University. Plasma samples from HCV patients with moderate to severe liver fibrosis and healthy control subjects (each group included 3 cases) were used for whole transcript digital gene expression (DGE) profiling sequencing. Other plasma samples were stored at −80°C and further detected by quantitative real-time polymerase chain reaction (qRT-PCR) analysis. Liver tissues were obtained by liver biopsy from 8 HCV patients with moderate to severe liver fibrosis among these HCV patients enrolled in this study, and normal liver tissues were obtained from 5 liver transplant donors. Part of liver tissue specimen was fixed in 10% formalin and subsequently processed for hematoxylin and eosin (H&E), Masson trichromatism, and immunohistochemical staining. Part of liver tissue specimen was frozen at −80°C for further analysis. Serum alanine aminotransferase (ALT) levels were measured by the Olympus biochemical autoanalyzer (AUS5400, Olympus Corporation, Tokyo, Japan).

**TABLE 1:** The demographical characters of subjects.

	**HC (*n* = 15)**	**HCV patients (*n* = 20)**	***t***	***P***
Age (years, mean ± SD)	51.2 ± 11.4	52.3 ± 7.4	−0.331	0.743
Gender (M/F, n)	9/6	11/9	–	–
ALT (U/L, mean ± SD)	28.9 ± 19.5	56.7 ± 39.8	−2.478	0.019
HCV RNA (lg IU/ml, mean ± SD)	–	6.2 ± 0.2	–	–
HCV genotype (n)	–			
1b	–	14		
2a		6		
Fibrosis stage	F0	F3-4		

### RNA Extraction and Purification

Total RNA of plasma was extracted using TRIzol reagent (Invitrogen, CA, United States) following the manufacturer’s procedure. The total RNA quantity and purity were analyzed of Agilent Bioanalyzer 2100 and RNA 6000 Nano LabChip Kit (Agilent Technologies, Santa Clara, CA, United States) with RIN number > 8.0. Approximately 5 μg of total RNA was used to deplete ribosomal RNA according to the manufacturer’s instructions of the Ribo-Zero^TM^ rRNA Removal kit (Epidemiology version, Epicenter, an Illumina company, Madison, WI, United States).

### mRNA Library Construction and Sequencing

Following purification, the mRNA was fragmented into small pieces using divalent cations under elevated temperature. Then the cleaved RNA fragments were reverse-transcribed to create the final DNA library in accordance with the protocol for the mRNA-Seq sample preparation kit (Illumina, San Diego, United States); the average insert size for the paired-end libraries was 300 bp (± 50 bp). And then we performed the paired-end sequencing (100 bp) on an Illumina Hiseq2500 at the LC-BIO (Hangzhou, China) following the vendor’s recommended protocol.

### Gene Function Analysis

The differentially expressed genes (defined as fold change > 2 and raw data *p* < 0.05) were input into the Database for Annotation, Visualization and Integrated Discovery (DAVID^1^) v6.7, which utilized GO to identify the molecular function represented in the gene profile and the Kyoto Encyclopedia of Genes and Genomes (KEGG) to analyze the potential functions of these genes in the pathways. The lower the *p*-value, the more significant the correlation is, and the recommended cut-off of *p*-value is 0.05.

### PCR Analysis

Total RNA was isolated from liver tissue and cell lines using TRIzol reagent (Invitrogen). First-strand cDNA was synthesized using a High Capacity cDNA Reverse Transcription kit (Applied Biosystems, Foster City, United States) according to instructions suggested by the manufacturer. Real-time PCR was performed using Power SYBR^®^ Green Master Mix (Applied Biosystems) and a StepOne^TM^ Real-Time PCR System (Applied Biosystems). Glyceraldehyde-3-phosphate dehydrogenase (GAPDH) gene expression was included as an internal control. The 2^–ΔΔCt^ method was used to calculate the relative expression of RNAs. Each assay was performed in triplicates and repeated three times, and the melt curve analysis and standard curves of each reaction were set up. The sequences of all primers used for qRT-PCR were listed in Table 2.

**TABLE 2:** The primer sequences.

**Gene names**	**Primer sequences (5′–3′)**	**Gene names**	**Primer sequences (5′–3′)**
ZFC3H1	F: CACATCCTGTGCCAAGAAGC	GRP78	F: TAGCGTATGGTGCTGCTGTC
	R: GCGAGATCACAGTTCGTGGA		R: TGACACCTCCCACAGTTTCA
GCLC	F: AAACCCAAACCATCCTACCC	EDEM1	F: ACCTGGCACGGGGCATGTTCG
	R: GCATGTTGGCCTCAACTGTA		R: GCGGCAGTGGATGGGGTTGAG
SLURP1	F: GTGACGGTGGAGGCAGAGTA	P58^IPK^	F: GCAGATACACAGATGCTACC
	R: TCGGAAGCAGCAGAAGATCA		R: AAACTTCAGAACAAACCCTAA
GUK1	F: GAACCTGTATGGCACGAGCA	CHOP	F: AACAGGCATCAGACCAGCTT
	R: AGATCGGTGGCCTTGATGTT		R: CCATCTCTGCAGTTGGATCA
IDH3G	F: ATGCCAACGTCATCCACTGT	IRE1	F: TAGTCAGTTCTGCGTCCGCT
	R: GCCACACTCTCATGCTCCAG		R: TTCCAAAAATCCCGAGGCCG
UBE2H	F: TTGAGTCCTTCCTGCCTCAGT	NF-κB	F: GAGCAATAGCCTGCCATGTT
	R: TTCTTCTGGTCGGTGGAGGT		R: GCCAATGAGATGTTGTCGTG
TMC5	F: CCATGTTCAGGCTTGTGGAG	IKKB	F: ACGACCTAGAGGAGCAAGCA
	R: CCACGGTGTTGAGCCAATAG		R: AGCTCTGAATTGCCTGAAGC
SCAMP3	F: ACCAGCCTATGAGCCTCCAG	TNFα	F: TCCTTCAGACACCCTCAACC
	R: GGCCTGAGTGCTGTATGAGC		R: CACATTCCTGAATCCCAGGT
PFKFB3	F: CGCGTACCATCTACCTGTGC	TGFβ1	F: GCAACAATTCCTGGCGATAC
	R: CCTCCACGAACTTGCTCAGA		R: CTAAGGCGAAAGCCCTCAAT
CMC1	F: CTCGACCCCGCAGTTCTAGT	MMP2	F: ATGACAGCTGCACCACTGAG
	R: GCTGGTTTGCTCCTGAAGACA		R: AGTTCCCACCAACAGTGGAC
α-SMA	F: CCCATCTATGAGGGCTATGC	TIMP1	F: ACATCCGGTTCGTCTACACC
	R: CACGCTCAGTCAGGATCTTC		R: TGATGTGCAAGAGTCCATCC
COL1	F: AGTGGTTTGGATGGTGCCAA	GAPDH	F: GGCATGGACTGTGGTCATGAG
	R: GCACCATCATTTCCACGAGC		R: TGCACCACCAACTGCTTAGC

### Immunohistochemistry (IHC)

IHC for α-smooth muscle actin (α-SMA), type I procollagen (COL1), and GCLC was performed on paraffin-embedded liver sections. Primary antibodies against α-SMA (1:200; ZSGB Biotechnology, Beijing, China), COL1 (1:100; Boster, Wuhan, China), and GCLC (1:100; Proteintech, Chicago, IL, United States) were used for immunoreaction. Negative controls were performed by replacing the specific primary antibody with PBS. The sections were incubated with horseradish peroxidase (HRP)-conjugated secondary antibody and developed with diaminobenzidine tetrahydrochloride substrate (DAB, ZSGB Biotechnology) for 1 min, counterstained briefly with hematoxylin, washed in PBS, dehydrated in graded alcohol, and mounted for microscopic analysis.

### Western Blot

Total proteins were extracted from liver tissues and cells using radio-immunoprecipitation buffer (RIPA). Equal amounts of denatured proteins were separated on 10% SDS-PAGE and analyzed by immunoblotting with primary antibodies against GCLC (1:1000; Proteintech, 12601-1-AP), α-SMA (1:1000; Proteintech, 14395-1-AP), COL1 (1:1000; Abcam, ab6308), glucose-regulated protein 78 (GRP78; 1:1000; Proteintech, 11587-1-AP), CCAAT/enhancer-binding protein homologous protein (CHOP; 1:1000; Proteintech, 15204-1-AP), phosphorylated inositol-requiring enzyme 1 (p-IRE1; 1:1000; Abcam, ab48187), endoplasmic reticulum degradation enhancer, mannosidase alpha-like 1 (EDEM1; 1:500; Proteintech, 26226-1-AP); protein kinase inhibitor (p58^IPK^; 1:1000; Abcam, ab70840); nuclear factor kappa B (NF-κB; 1:1000; Proteintech, 14220-1-AP), inhibitor of KB kinase b (IKKB; 1:1000; Proteintech, 15649-1-AP), tumor necrosis factor α (TNFα; 1:1000; Abcam, ab6671), transforming growth factor 1 (TGFβ1; 1:1000; Proteintech, 21898-1-AP), tissue inhibitor of the metalloproteinase (TIMP1; 1:1000; Proteintech, 16644-1-AP), Matrix metalloproteinase 2 (MMP2; 1:1000; Proteintech, 10373-2-AP), and β-actin (1:10000; Proteintech, 14395-1-AP). After the incubation with HRP-conjugated secondary antibodies, the signals were visualized by the ECL system (Thermo Fisher Scientific).

### Establishment of Stable Cell Lines With HCV Core Protein Infection

Plasmid pcDNA3.1 (−) −HCV-core, which contains the complete coding region of the HCV-core protein (Genotype 1b), and the control plasmid pcDNA3.1 (−) −NC was generously provided as gifts by Professor Jun Cheng (Institute of Infectious Diseases, Beijing Ditan Hospital, Capital Medical University, China). LO2 cells stably transfected with HCV-core protein (named LO2-CORE) and the control cells (named LO2-NC) were established and verified according to the method described previously (Fu et al., 2016). The culture supernatants derived from LO2-CORE and LO2-NC cells were collected at 5 days. LO2 cell line was usually used as the normal human hepatocytes, but actually there was evidence that LO2s might in fact be compromised by HeLa cells (Ye et al., 2015). So we also used HepG2-CORE and HepG2-NC cells which we constructed in our previous study (Fu et al., 2016) as the second cell line to increase the reliability of the study.

### Cell Culture and Treatment

Human HSC cell line (LX-2) were chosen and cultured in our study. LX-2 were gifted by prof. Xu Lieming from Shuguang Hospital of Shanghai University of Traditional Chinese Medicine. The cells were cultured in Dulbecco’s modified Eagle’s medium (DMEM) (Hyclone, Logan, Utah, United States) supplemented with 10% fetal bovine serum (FBS) (BI, Kibbutz Beit Haemek, Israel), penicillin (100 U/ml), and streptomycin (100 μI/ml), and incubated at 37°C at an atmosphere of 5% CO_2_. To activate LX-2 cells, LX-2 cells were treated with culture supernatant derived from LO2-CORE and LO2-NC cells or HepG2-CORE and HepG2-NC cells (constructed in our previous study) for 48 h. LX-2 cells were collected at 48 h for RT-PCR and Western blot assay. All experiments were repeated three times with triplicate measurements for each assay.

### Plasmid Transfection and L-Buthionine-Sulfoximine (L-BSO) Treatment

LX-2 cells were seeded in a 6-well plate at a density of 2 × 10^5^/ml with DMEM medium without antibiotics and cultured for 24 h before transfection. Four micrograms of puc19-hGCLC and puc19 plasmid (Sino Biological Inc., Beijing, China) were transfected into LX-2 cells using Lipofectamine 2000 for overexpression of GCLC. The culture medium was replaced with fresh medium or medium containing L-BSO (500 μmol/l) after 6 h. LX-2 cells were collected at 48 h for further analysis. All experiments were repeated three times with triplicate measurements for each assay.

### GSH Detection

The concentration of GSH in the supernatant was determined by using spectrophotometric diagnostic kits (Nanjing Jiancheng Biotechnology Institute) according to the manufacturer’s protocol, which uses 5, 5-dithio-bis-2-nitrobenzoic acid (DTNB) for color development. The yellow color developed was monitored at 405 nm on a spectrophotometer. The GSH, GSSG concentration, and the ratio of 2GSH/GSSG was calculated according to the manufacturer’s protocol.

### Intracellular Reactive Oxygen Species (ROS) Measurement

Intracellular ROS levels were measured as described previously using dihydrodichlorofluorescein diacetate (DCFH-DA, Nanjing Jiancheng Biotechnology Institute, China), a cell-permeable dye that becomes fluorescent upon oxidation by ROS. After treatment LX-2 cells were incubated with 10 μM DCFH-DA in PBS for 30 min at 37°C. Cells were washed twice with ice-cold PBS and suspended by trypsinization, harvested by centrifugation (4°C, 3000 rpm, 5 min), resuspended in 1 ml PBS, and finally analyzed by flow cytometry (FCM) with 488 nm excitation and 525 nm emission detection. Values of cellular fluorescence were obtained using FCS Express Version 3.

### Statistical Analysis

Data were presented as mean ± SD and analyzed using SPSS 16.0 software package. Difference between two groups was analyzed using the Student *t*-test, and the difference among multiple groups was evaluated using one-way analysis of variance, with the least significant difference-*t*-test (LSD-*t*) for *post hoc* comparison. All *P*-values were two-tailed and were considered significant at *P* < 0.05.

## Results

### Patient Characteristics

Characteristics of patients with HCV-related liver fibrosis and healthy control subjects including Age, Sex, serum ALT, HCV genotype, HCV-RNA load, and fibrosis stage of liver sample were listed in Table 1. Serum ALT levels were increased in patients with HCV-related fibrosis compared with healthy controls.

### Digital Gene Expression (DGE) Profiles and Validated by qRT-PCR

**FIGURE 1:**
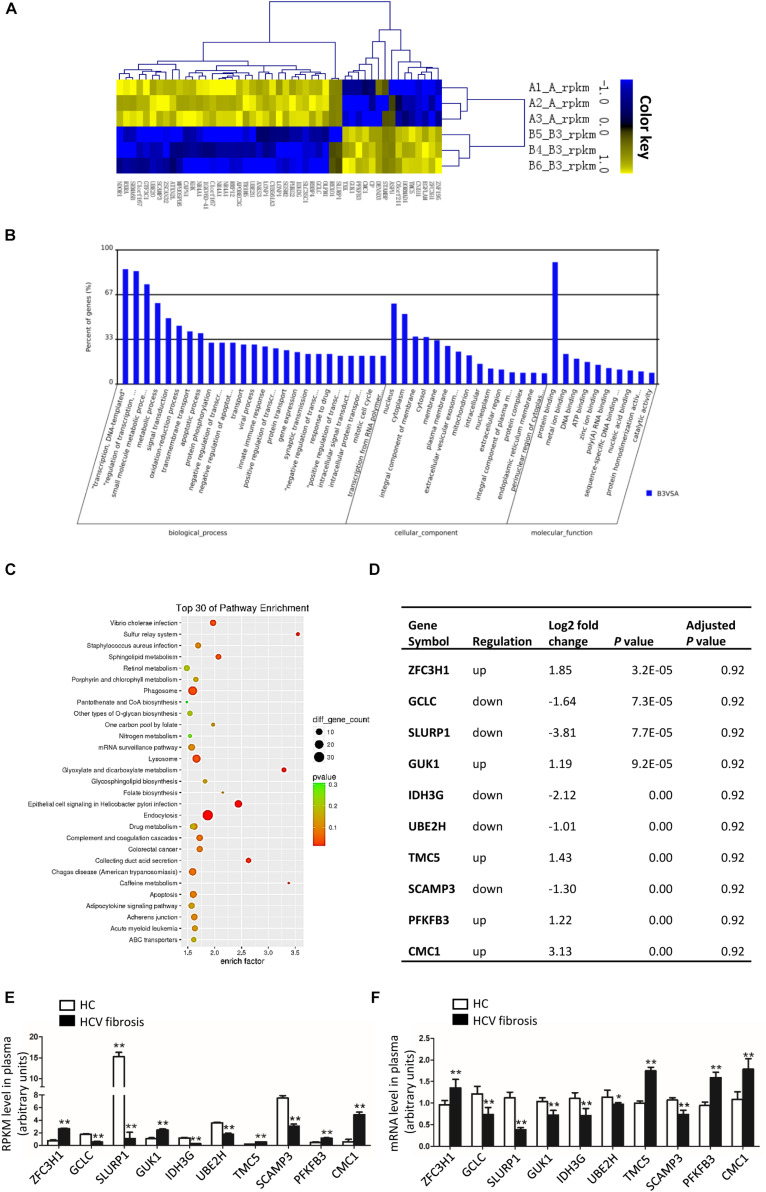
Differentially expressed genes in patients with HCV-related liver fibrosis. **(A–C)** Heat map **(A)**, GO analysis **(B)**, and KEGG pathway analysis **(C)** of the differentially expressed genes of patients with HCV-related liver fibrosis. **(D)** List of top 10 differentially expressed genes in patients with HCV-related liver fibrosis according to the *P-*value of raw data. And the *P*-values were adjusted by Benjamini-Hochberg method to correct the false discovery rate. **(E)** RPKM level of top 10 genes in plasma. **(F)** mRNA levels of top 10 genes in plasma from Healthy control (*n* = 15) and HCV-related liver fibrosis patients (*n* = 20) were detected by qRT-PCR. *P < 0.05, **P < 0.01. HC, healthy control.

According to the results of gene sequencing, we identified that 622 mRNAs were up-regulated and 1291 mRNAs were down-regulated in patients with HCV-related liver fibrosis. The heatmap in Figure 1A showed the TOP 50 genes of differentiated expressed. GO analysis in Figure 1B showed the main biological process of the differentially expressed genes was transcription, the cellular component was nuclear and cytoplasm, and the molecular function was protein binding. KEGG Pathway enrichment analysis (Figure 1C) showed the enriched pathways including epithelial cell signaling in Helicobacter pylori infection, endocytosis, lysosome, and Glyoxylate and dicarboxylate metabolism. The top 10 differentially genes were zinc finger C3H1-type containing (ZFC3H1), GCLC, secreted LY6/PLAUR domain containing 1 (SLURP1), guanylate kinase 1 (GUK1), isocitrate dehydrogenase 3 [NAD(+)] gamma (IDH3G), ubiquitin conjugating enzyme E2 H (UBE2H), transmembrane channel like 5 (TMC5), secretory carrier membrane protein 3 (SCAMP3), 6-phosphofructo-2-kinase/fructose-2,6-biphosphatase 3 (PFKFB3), and C-X9-C motif containing 1 (CMC1) (Figure 1D), and GCLC as the antioxidative gene might participate in the pathogenesis of HCV-related liver fibrosis. As shown in Figures 1E,F, the tendency of mRNA expression of top 10 differentially expressed genes detected by qRT-PCR was consistent with the reads per kilobase per million mapped reads (RPKM) level from sequencing data.

### GCLC Was Dramatically Downregulated in the Liver of Patients With HCV-Related Hepatic Fibrosis

**FIGURE 2:**
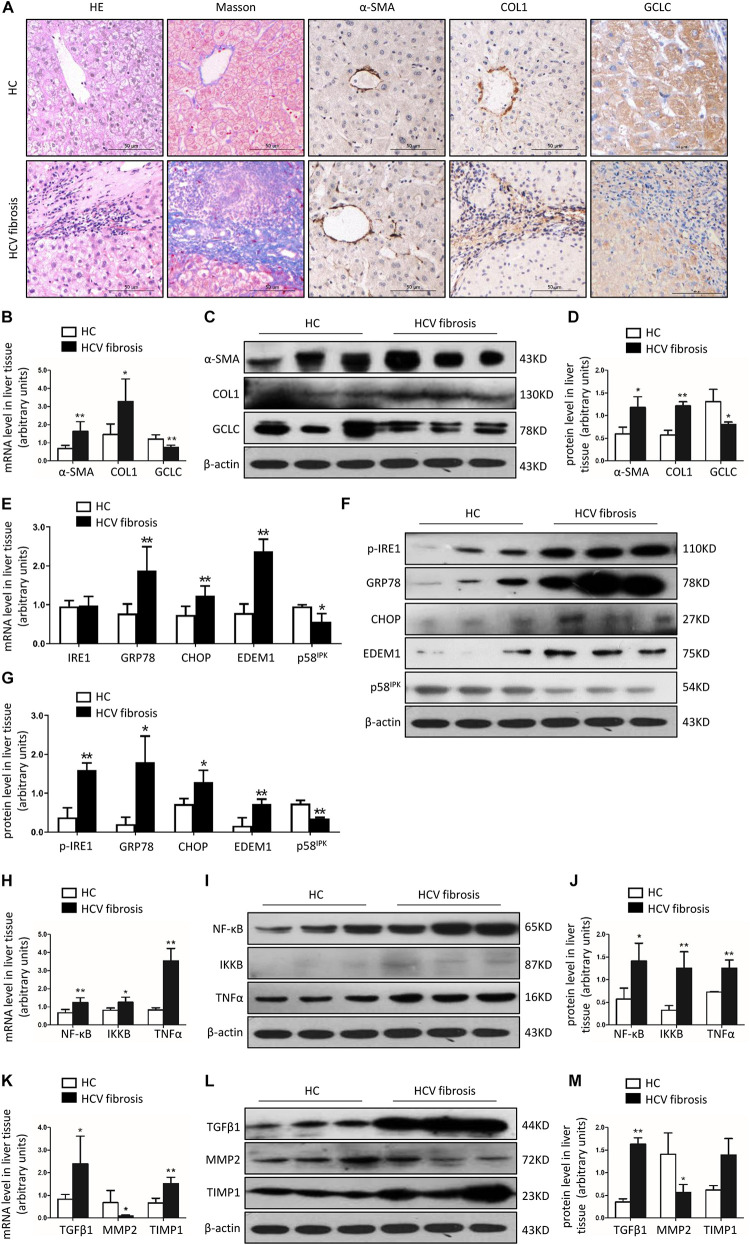
GCLC was dramatically decreased in liver tissues of patients with HCV-related liver fibrosis. Liver tissues from healthy liver transplant donor (*n* = 5) and patients with HCV-related liver fibrosis (*n* = 8) were detected. **(A)** Liver fibrosis was evaluated with Masson staining, and expression of α-SMA, COL1, and GCLC were detected by IHC. Positive staining was indicated by brown color. **(B–D)** Expression of α-SMA, COL1, and GCLC in liver tissues of HCV patients was detected by qRT-PCR **(B)** and Western blot **(C,D)**. **(E–G)** Expression of ER stress-related genes in liver tissues of HCV patients was detected by qRT-PCR **(E)** and Western blot **(F,G)**. **(H–J)** Expression of inflammatory factor NF-κB, IKKB, and TNFα in liver tissues of HCV patients was detected by qRT-PCR **(H)** and Western blot **(I,J)**. **(K–M)** Expression of fibrotic genes such as TGFβ1, MMP2, and TIMP1 in liver tissues of HCV patients was detected by qRT-PCR **(K)** and Western blot **(L,M)**. *P < 0.05, **P < 0.01. HC: healthy control.

Patients with HCV-related hepatic fibrosis showed increased serum ALT levels and liver fibrosis degree (Table 1), accompanied by a parallel increase in the expression of hepatic α-SMA and COL1, and a dramatically decreased expression of hepatic GCLC (Figure 2A). Also, the RT-PCR and Western blot analysis verified an increase in α-SMA and COL1 expression and a decrease in GCLC mRNA (Figure 2B) and protein expression (Figures 2C,D) in HCV-related hepatic fibrosis. Furthermore, we detected the expression of ER stress-related genes, including branches of unfolded protein response (UPR) and the target gene of UPR, such as IRE1, GRP78, CHOP, EDEM1, and p58^IPK^. The results showed that the mRNA (Figure 2E) and protein expression (Figures 2F,G) of p-IRE1, GRP78, CHOP, and EDEM1 were notably up-regulated, and p58^IPK^ mRNA (Figure 2E) and protein expression (Figures 2F,G) was decreased in liver tissue of patients with HCV-related hepatic fibrosis, which indicated that ER stress was provoked in the progression of HCV-induced liver fibrosis. We further detected downstream inflammatory factors and profibrogenic genes. In patients with HCV-related hepatic fibrosis, NF-κB, IKKB, and TNFα mRNA (Figure 2H) and protein were highly increased (Figures 2I,J). Also, TGFβ1 and TIMP1 were up-regulated, however, MMP2 was dramatically decreased compared to healthy control at mRNA (Figure 2K) and protein level (Figures 2L,M).

### GCLC Was Decreased in Activated HSC Induced by Condition Medium From LO2-CORE and HepG2-CORE

**FIGURE 3:**
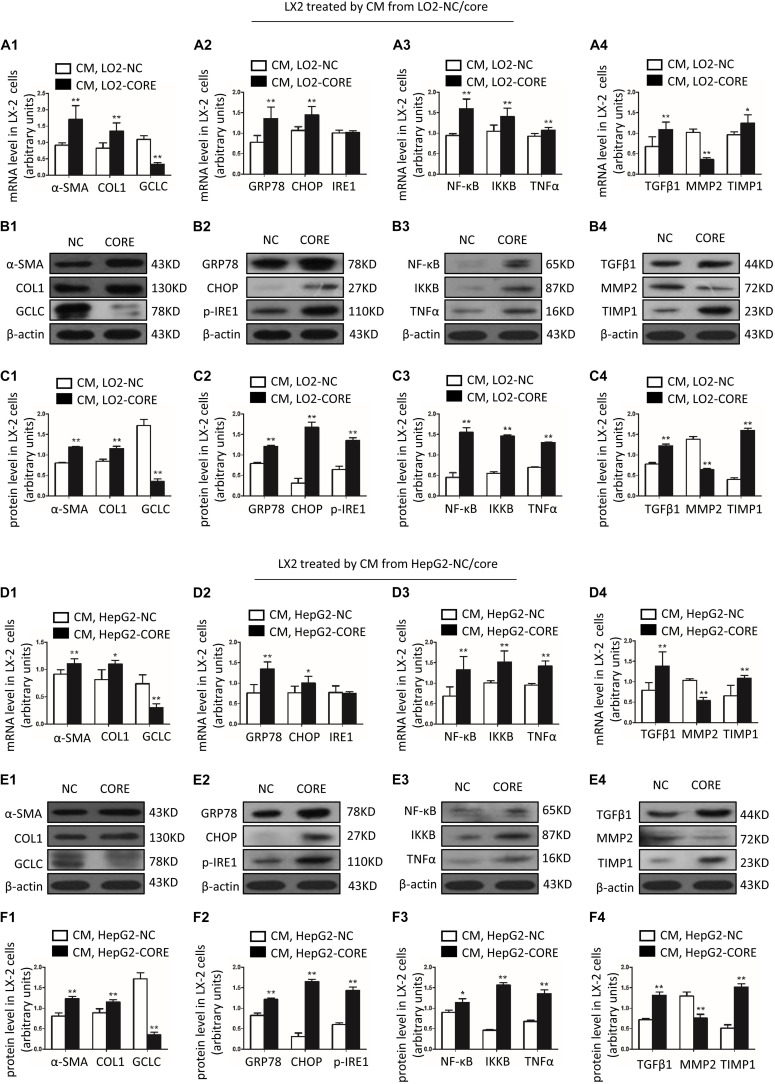
GCLC was decreased in activated HSC induced by condition medium from LO2-CORE and HepG2-CORE. **(A1–C1)** Expression of α-SMA, COL1, and GCLC in activated HSC induced by CM from LO2-CORE was detected by qRT-PCR **(A1)** and Western blot **(B1,C1)**. **(A2–C2)** Expression of ER stress-related genes GRP78, CHOP, and IRE1 in activated HSC was detected by qRT-PCR **(A2)** and Western blot **(B2,C2)**. **(A3–C3)** Expression of inflammatory factor NF-κB, IKKB, and TNFα in activated HSC was detected by qRT-PCR **(A3)** and Western blot **(B3,C3)**. **(A4–C4)** Expression of fibrotic genes such as TGFβ1, MMP2, and TIMP1 in activated HSC was detected by qRT-PCR **(A4)** and Western blot **(B4,C4)**. **(D1–F1)** Expression of α-SMA, COL1, and GCLC in activated HSC induced by CM from HepG2-CORE was detected by qRT-PCR **(D1)** and Western blot **(E1,F1)**. **(D2–F2)** Expression of ER stress-related genes in activated HSC was detected by qRT-PCR **(D2)** and Western blot **(E2,F2)**. **(D3–F3)** Expression of inflammatory factors in activated HSC was detected by qRT-PCR **(D3)** and Western blot **(E3,F3)**. **(D4–F4)** Expression of fibrotic genes in activated HSC was detected by qRT-PCR **(D4)** and Western blot **(E4,F4)**. *P < 0.05, **P < 0.01. CM: conditioned medium.

We constructed LO2-CORE cell line by transfecting HCV core plasmid to LO2 cell line. LO2-CORE cell could express HCV core protein stably (Supplementary Figures S1A,B); the supernatant from LO2-CORE cell as condition medium (CM), which contained a gradual increased TGF β1 level (Supplementary Figure S1C), was used to treated LX-2 cells. It showed that α-SMA and COL1 mRNA (Figure 3A1) and protein were dramatically increased (Figures 3B1,C1), which indicated that LX-2 could be activated by the CM from LO2-CORE cells. We further detected the expression of GCLC in activated HSCs; as shown in Figures 3A1–C1, GCLC mRNA and protein were notably downregulated in activated HSCs. The results demonstrated that GCLC was a negative regulation in the progression of the activation of HSCs. And in the activated HSCs, ER stress-related gene GRP78, CHOP was upregulated in mRNA (Figure 3A2) and protein level (Figures 3B2,C2), and p-IRE1 was increased at protein level (Figures 3B2,C2). Also, the inflammatory factor NF-κB, IKKB, and TNFα (Figures 3A3–C3), and profibrogenic genes TGFβ1, TIMP1 was dramatically increased (Figures 3A4–C4) in activated HSCs compared with the control group. Then we repeated the detection of these relative genes in activated HSCs induced by condition medium from HepG2-CORE, which was set up as a cell model for HCV-related hepatic fibrosis according to our previous study (Huang et al., 2017). The results showed similar tendency changes. The LX-2 cells were treated with CM from HepG2-Core cells, which was indicated by up-regulated α-SMA and COL1, and companied with down-regulated GCLC (Figures 3D1–F1). Also, ER stress-related gene GRP78, CHOP was upregulated in activated HSCs, while p-IRE1 was increased at protein level (Figures 3D2–F2). Meanwhile, NF-κB, IKKB, and TNFα (Figures 3D3–F3), and profibrogenic genes TGFβ1, TIMP1 was dramatically increased (Figures 3D4–F4).

### Overexpression of GCLC Could Suppress the Activation of HSC and Regulate the Expression of Profibrogenic Factor

**FIGURE 4:**
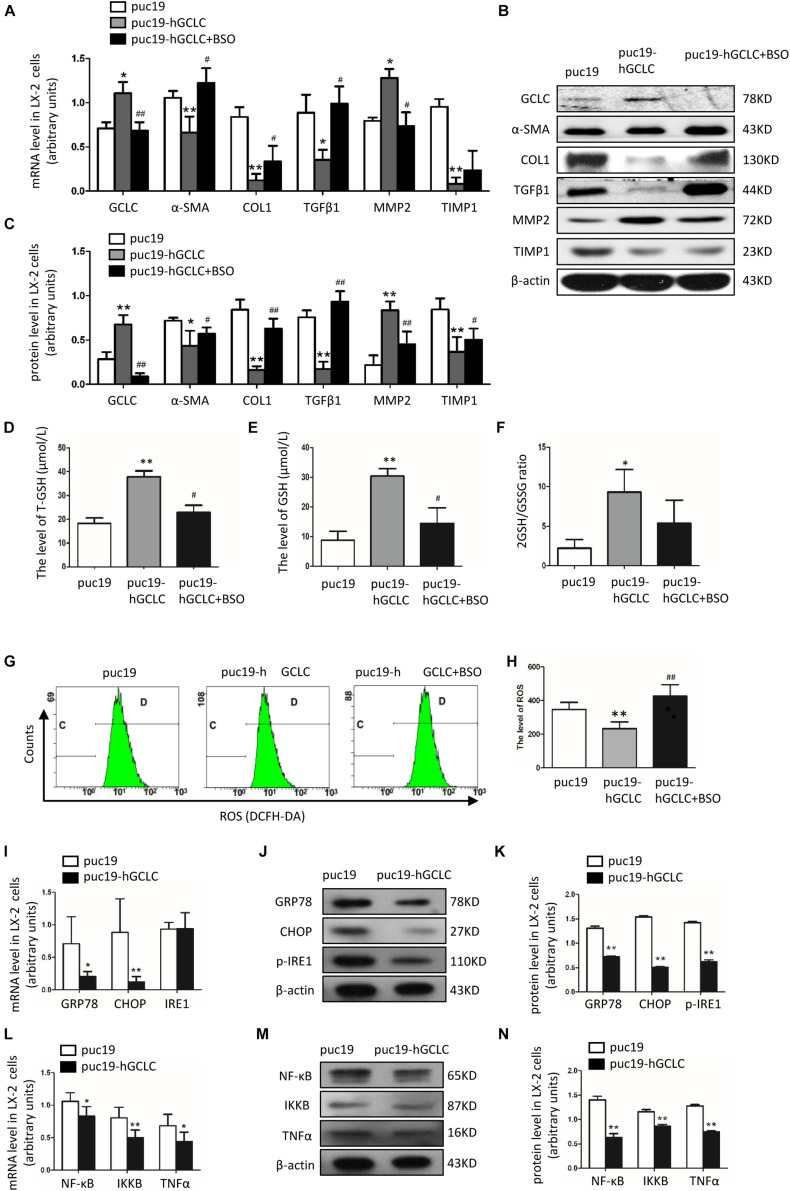
Overexpression of GCLC could suppress the activation of HSC. **(A–C)** Expression of GCLC, α-SMA, COL1, TGFβ1, MMP2, and TIMP1 in LX-2 cells transfected by puc19, puc19-hGCLC, and GCLC-expressing LX-2 cells treated with L-BSO was detected by qRT-PCR **(A)** and Western blot **(B,C)**. **(D–F)** The level of total GSH **(D)**, GSH **(E)**, and 2GSH/GSSG ratio **(F)** was increased in LX-2 cells transfected with the puc19-hGCLC plasmid. **(G–H)** ROS-DCF was dramatically decreased in LX-2 cells transfected with the puc19-hGCLC plasmid. **(I–K)** Expression of ER stress-related gene GRP78, CHOP, and IRE1 in LX-2 cells transfected with puc19 and puc19-hGCLC plasmid was detected by qRT-PCR **(I)** and Western blot **(J,K)**. **(L–N)** Expression of inflammatory factors NF-κB, IKKB, and TNFα mRNA and protein was detected by qRT-PCR **(L)** and Western blot **(M,N)**. *P < 0.05, **P < 0.01; ^#^*P* < 0.05, ^##^*P* < 0.01.

In order to overexpress GCLC in LX-2, we transfected puc19-NC and puc19-hGCLC to LX-2 cell, and verified with GCLC mRNA and protein detection, which showed GCLC was overexpressed in LX-2 cell transfected with puc19-hGCLC (Figures 4A–C). Compared to puc19-NC group, LX-2 cell overexpressed GCLC could turn into quiescent phenotype, as indicated by reduced expression of α-SMA and COL1 at mRNA (Figure 4A) and protein level (Figures 4B,C). Meanwhile, the profibrogenic factor TGFβ1 and tissue inhibitor of metalloproteinase (TIMP1) were down-regulated in LX-2 transfected by puc19-hGCLC, however, MMP2 was dramatically increased compared to LX-2 by transfected puc19-NC (Figures 4B,C). We then used L-BSO, a compound that affects GSH biosynthesis, to inhibit the GSH production (Figures 4D–F) and found that L-BSO could overt the suppress effects of GCLC in LX-2, which was indicated by increased α-SMA, COL1, TGFβ1, and TIMP1, also decreased MMP2 (Figures 4A–C).

### Overexpression of GCLC Could Increase GSH Level and Decrease ROS Level

Because GCLC could regulate the synthesis of GSH, we further detected GSH and ROS content in order to illustrate the effect of overexpression of GCLC in LX-2 cells. The total GSH and GSH levels were highly increased in LX-2 cells transfected by puc19-hGCLC compared to that in LX-2 cells transfected by puc19 (Figures 4D,E). And 2GSSH/GSH ratio was notably decreased when LX-2 cells were overexpressed GCLC (Figure 4F). And L-BSO could suppress the product of total GSH and GSH (Figures 4D,E). Also, the intracellular ROS level was detected with DCFH-DA, and the results showed ROS level in GCLC-overexpressing LX-2 cells was notably decreased (Figures 4G,H), and L-BSO could block this effect and lead to increased ROS (Figures 4G,H).

### Overexpression of GCLC Could Inhibit ER Stress and Its Downstream Inflammatory Factor

ROS could promote ER stress and inflammatory factor release, leading to the progression of fibrosis. When overexpressed GCLC in LX-2 cells, we found that ROS level was dramatically decreased (Figures 4G,H), concomitantly with a dramatically decreased expression of ER stress-related genes. It showed that GRP78, CHOP, and p-IRE1 were all decreased in LX-2 cells transfected by puc19-hGCLC (Figures 4I–K). The downstream inflammatory factors such as NF-κB, IKKB, and TNFα were also down-regulated compared to that in LX-2 cells transfected by puc19 (Figures 4L–N).

## Discussion

In this study, we explored the differentially expressed mRNAs between patients with HCV-related liver fibrosis and healthy control subjects. GCLC, known as an important antioxidative gene (Ramani et al., 2012), was verified dramatically decreased in plasma and liver tissue of patients with HCV-related liver fibrosis, and also it was down-regulated in activated HSC induced by conditioned medium from HCV core stably expressed cell lines, LO2-CORE and HepG2-CORE. The results indicated that GCLC might participate in the pathogenesis of HCV-related liver fibrosis. Meanwhile, with the progression of hepatic fibrosis and the activation of HSC, the expression of ER stress-related genes and ER stress-induced inflammatory factors and profibrogenic factors were highly increased. Furthermore, we explored the relationship between GCLC and ER stress, inflammation, and fibrosis through gain-of-function experiments. Overexpression of GCLC in LX-2 cells could increase GSH level and decrease ROS production, which suppressed ER stress and reduced hepatic inflammation and fibrosis, and inhibition of GSH biosynthesis by L-BSO could overt the effect of GCLC on liver fibrosis. It demonstrated that GCLC was a negative regulatory factor of the activation of HSC. To our knowledge, our data provided the evidence that GCLC might suppress HSC activation and ameliorate HCV-related hepatic fibrosis and suppress ER stress.

Emerging evidence showed that GCLC was involved in the pathogenesis of liver fibrosis. GCLC^h/h^ mice with a hepatocyte-specific ablation of GCLC developed severe steatosis, although with L-N-acetylcysteine treatment, GCLC^h/h^ mice also display characteristics of fibrosis at age 50 days, and even macronodular cirrhosis at age 120 days (Chen et al., 2010). Another report showed knockdown of GCLC could exacerbate bile duct ligation-induced liver injury and fibrosis; moreover, the anti-fibrotic effect of ursodeoxycholic acid and S-adenosylmethionine was nearly lost when GCLC induction was blocked (Ramani et al., 2012). And overexpression of GCLC *in vivo* could significantly decrease collagens in CCL4-induced and pig serum-induced liver fibrosis model (Wang et al., 2016). In our study, we first found that GCLC was dramatically downregulated in plasma and liver tissues of patients with HCV-related liver fibrosis and activated HSC induced by conditioned medium from LO2-core and HepG2-core cells, which indicated that GCLC negatively regulated the progression of HCV-induced liver fibrosis and the activation of HSC. Then we further explored the potential mechanism of GCLC in the development of HCV-related liver fibrosis.

As we know, oxidative stress was a major response for HCV infection (Fujinaga et al., 2011). HCV core protein enhanced the expression of mitochondrial respiratory enzyme chaperone and promoted ROS production through electron leakage from mitochondrial electron transport, resulting in mitochondrial damage (Machida et al., 2004). Also, HCV could directly increase the production of superoxide and H2O2 in hepatocytes by upregulating the expression of nicotinamide adenine dinucleotide phosphate oxidase (Nox) protein, promoting mitochondria ROS generation and decreasing GSH synthesis (Boudreau et al., 2009; de Mochel et al., 2010; Choi et al., 2014). On the other hand, HCV infection also hampered antioxidant systems in the liver. HCV could blunt Nrf2 activation and inhibit antioxidative responsive element (ARE)- regulated gene expression (Carvajal-Yepes et al., 2011), thereby decreased the antioxidant role of the Nrf2/ARE pathway (Ivanov et al., 2011). Thus, the imbalance of oxidant/antioxidant state in the liver contributed to the pathogenesis of HCV-related liver diseases (Torok, 2016). GCLC was a key gene of GSH synthesis; it could be activated by nuclear factor erythroid-2-related factor 2 (Nrf2)-Keap signal pathway (Shi et al., 2016), which also could activate the transcription of heme oxygenase-1 (HO-1) and NAD(P)H: quinone oxidoreductase-1 (NQO1). Nrf2-GCLC system has been considered as the key antioxidative stress system in the liver (Dornas et al., 2019; Paunkov et al., 2019). And in patients with HCV-related liver fibrosis, GCLC was notably decreased in plasma and liver tissue, and it also decreased in activated HSC induced by conditioned medium derived from LO2-core and HepG2-core cell lines, which indicated that antioxidant role of GCLC was disturbed. Moreover, ROS as the major production of oxidative stress (Cichoz-Lach and Michalak, 2014) were evidenced to induce the proliferation, migration of HSCs, and the production of collagen (Sanchez-Valle et al., 2012). And overexpression of GCLC *in vivo* could decrease the ROS production and increase the level of GSH, which was the major antioxidant in the liver and could maintain the quiescent phenotype of HSC, and the inhibitor of GSH synthesis could temper these changes.

These results indicated that GCLC could inhibit the activation of HSC by regulating the oxidize-redox system in the liver. Thus, antioxidative stress therapy through overexpressing GCLC might be a new treatment target of HCV-related liver fibrosis.

Furthermore, ER stress was characterized by the accumulation of misfolded proteins (also known as the UPR) (Adams et al., 2019), and it could drive inflammatory signaling (Duvigneau et al., 2019) and be involved in the progression of liver disease (Loeuillard et al., 2018), including HCV-related liver disease (Chusri et al., 2016). UPR consisted of three branches of stress sensors: ATF6, IRE1-XBP1 and PERK, and UPR target gene included GRP78, CHOP, EDEM1, and p58^IPK^ et al. (Loeuillard et al., 2018). These UPR target genes were notably increased in liver tissues from HCV patients with liver fibrosis, which was supported by previous findings (Koo et al., 2016). Moreover, ER stress could induce hepatocyte apoptosis to promote liver fibrosis via stimulator of interferon genes (STING) and interferon regulatory factor 3 (IRF3) (Iracheta-Vellve et al., 2016), which could explain why the pro-apoptosis gene CHOP was highly expressed in HCV patients with liver fibrosis. Also, ER stress was evidenced to contribute to HSC activation; the possible regulatory mechanism was that ER stress in HSCs could promote liver fibrosis by inducing overexpression of SMAD2, via dysregulation of miR18a, and this dysregulation was mediated by PERK phosphorylation and destabilization of HNRNPA1 (Koo et al., 2016). With our findings, ER stress-related genes were increased in activated HSC, and overexpression of GCLC in LX-2 cells could suppress the expression of ER stress markers, which demonstrated that GCLC could inhibit ER stress. However, CHOP as a pro-apoptosis gene was also increased in activated HSC, and overexpression of GCLC could decrease CHOP expression, which might contribute to the apoptosis of activated HSC, leading to ameliorate liver fibrosis. Although these results could be supported by several studies, De Minicis et al. (2012) had evidenced that ER stress could induce HSC apoptosis and contributed to fibrosis resolution. It meant that ER stress might play two opposing effects on liver fibrosis, including adaptation and apoptosis. ER stress-induced apoptosis of hepatocytes might be profibrogenic to activate HSC by paracrine mechanism, while the apoptosis of HSCs would be expected to be fibrogenic resolution (Li et al., 2015).

In addition, oxidative stress and ER stress had been evidenced to promote the progression of inflammation and fibrosis in the liver (Son et al., 2009). HCV regulated hepatic fibrosis progression through inducing ROS generation and NF-κB activation (Salloum et al., 2016). HCV non-structural protein 5A (NS5A) was also engaged in the ER stress, and it ultimately lead to the activation of STAT-3 and NF-κB (Cheng et al., 2015). Also, HCV core protein could increase cell proliferation of HSC in a Ras/ERK and PI3K/AKT dependent manner (Nakamura et al., 2011). HCV core and NS protein induced proinflammatory actions, such as increased chemokine secretion and ICAM-1 expression through NF-κB/C-Jun N-terminal kinase pathways (Bataller et al., 2004). As in our research, NF-κB might be activated under ER stress condition and was highly increased in patients with HCV-related liver fibrosis. NF-κB as a nuclear factor could further activate TNFα and COX-2 (Lu et al., 2015), which could increase the secretion of TGFβ1, leading to increased expression of α-SMA and COL1, contaminant with upregulating the expression of TIMP1 and decreasing the expression of MMP2. And overexpression of GCLC could dramatically attenuate inflammation and fibrosis through regulating proinflammatory and profibrogenic genes.

In conclusion, GCLC is a negative regulatory factor in the progression of HCV-related liver fibrosis. And overexpression of GCLC may ameliorate the development of liver fibrosis in HCV patients through decreasing ROS production, increasing the GSH level, and also attenuate ER stress and inflammation. Hence, GCLC may function as a novel anti-fibrosis factor and be a potential therapeutic target for HCV-related liver fibrosis.

## Data Availability Statement

The raw data generated by this study can be found in the GEO, accession number GSE154055.

## Ethics Statement

The studies involving human participants were reviewed and approved by the Human Ethics Committee of the Third Hospital of Hebei Medical University No. 1/016/2014. The patients/participants provided their written informed consent to participate in this study.

## Author Contributions

NF and DL contributed to the study design, statistical analysis, and manuscript writing. WL and WZ participated in the collection of clinical data and samples. SiZ and LL helped in finish the laboratory work. SuZ, JD, and LK participated in the analysis of data. RW, YZ, and YN took responsibility for study design and critical revision of the manuscript. All authors read and approved the final manuscript.

## Conflict of Interest

The authors declare that the research was conducted in the absence of any commercial or financial relationships that could be construed as a potential conflict of interest.

## Footnotes

1 http://david.abcc.ncifcrf.gov/

## Supplementary Material

FIGURE S1 HCV core protein-expressing LO2-CORE cell line was set up. **(A,B)** HCV core protein was detected by qRT-PCR **(A)** and Western blot **(B)** in LO2 cells transfected by plasmid pcDNA3.1 (−) −HCV-core and pcDNA3.1 (−) −NC. **(C)** TGFβ1 level in cell supernatants of HCV CORE-expressing LO2 cells and control LO2 cells was detected by ELISA. **P* < 0.05, ***P* < 0.01.
