# Supplementary figures and images for: Glutamate-Cysteine Ligase Catalytic Subunit Attenuated Hepatitis C Virus-Related Liver Fibrosis and Suppressed Endoplasmic Reticulum Stress
Source: Front Mol Biosci. 2020 Aug 18;7:199. doi: 10.3389/fmolb.2020.00199 (PMC7461853; doi:10.3389/fmolb.2020.00199)

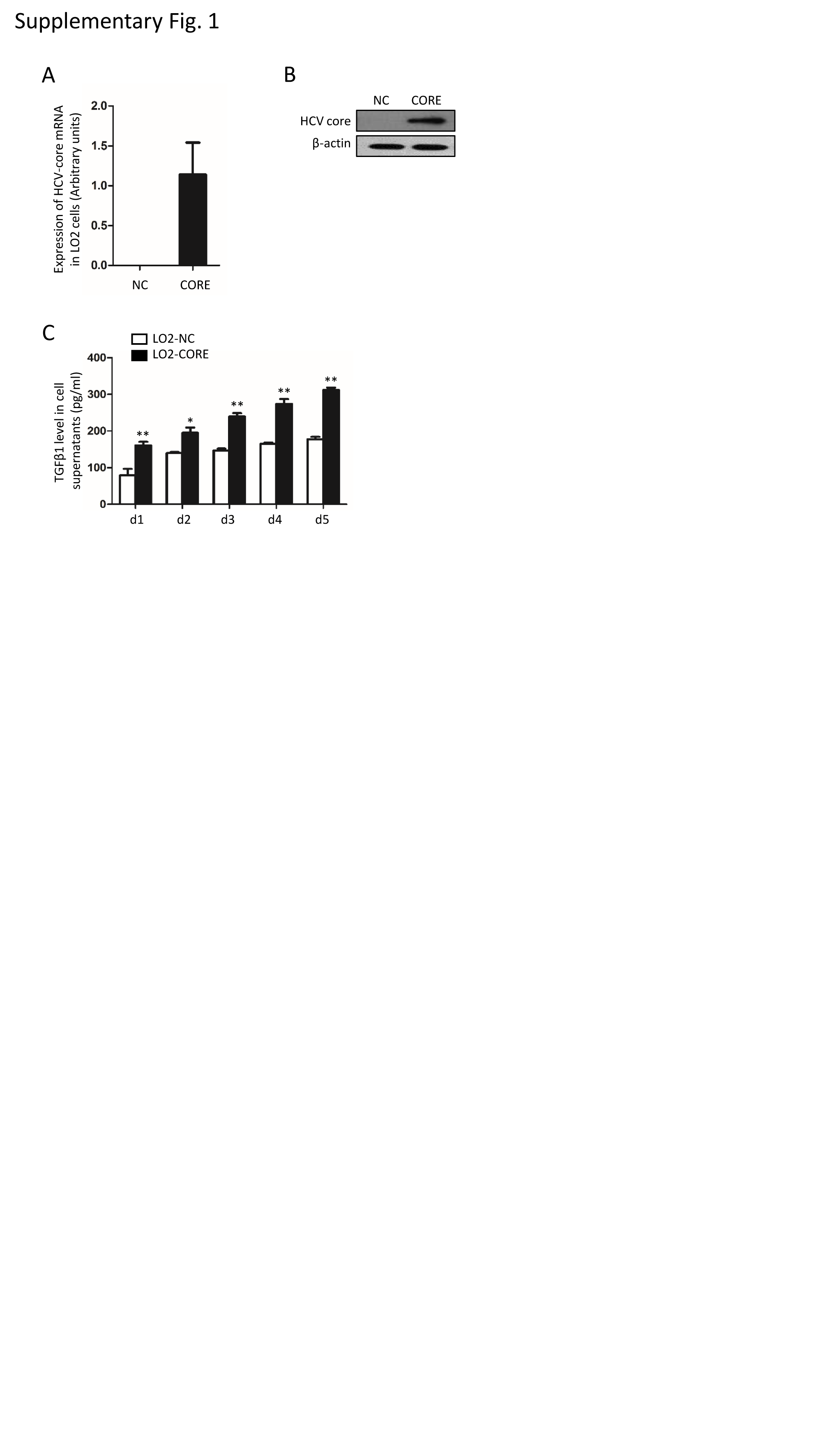

Supplement: FIGURE S1 — HCV core protein-expressing LO2-CORE cell line was set up. (A,B) HCV core protein was detected by qRT-PCR (A) and Western blot (B) in LO2 cells transfected by plasmid pcDNA3.1 (−) −HCV-core and pcDNA3.1 (−) −NC. (C) TGFβ1 level in cell supernatants of HCV CORE-expressing LO2 cells and control LO2 cells was detected by ELISA. *P < 0.05, **P < 0.01. [file Image_1.TIF]
